# Influencing factors on instrumental activities of daily living functioning in people with mild cognitive disorder – a secondary investigation of cross-sectional data

**DOI:** 10.1186/s12877-022-03476-8

**Published:** 2022-10-11

**Authors:** Marina Bruderer-Hofstetter, Ellen Gorus, Elise Cornelis, André Meichtry, Patricia De Vriendt

**Affiliations:** 1 School of Health Professions, Institute of Physiotherapy, ZHAW Zurich University of Applied Sciences, Katharina-Sulzer-Platz 9, CH-8400 Winterthur, Switzerland; 2 Department Gerontology and Frailty in Ageing (FRIA) Research Group, Vrije Universiteit Brussel (VUB), Brussels, Belgium; 3 Geriatrics Department, Universitair Ziekenhuis Brussel (UZ Brussel), Brussels, Belgium; 4 Department of Occupational Therapy and Research & Development in Health & Care, Artevelde University of Applied Sciences, Ghent, Belgium; 5 Department Gerontology and Frailty in Ageing (FRIA) Research Group, Mental Health and Wellbeing (MENT) Research Group, Vrije Universiteit Brussel (VUB), Brussels, Belgium; 6 Department of Occupational Therapy, Artevelde University of Applied Sciences, Ghent, Belgium; 7 Faculty of Medicine and Health Sciences, Department of Rehabilitation Sciences and Physiotherapy, Occupational Therapy Programme, Ghent University, Ghent, Belgium

**Keywords:** Mild neurocognitive disorder, Instrumental activities of daily living, Cognitive function, Physical function, Personal and environmental factors

## Abstract

**Background:**

Finding a strategy to reduce the impact of cognitive decline on everyday functioning in persons suffering from cognitive impairment is a public health priority. Instrumental activities of daily living (IADL) are key to everyday functioning. Hence, it is essential to understand the influencing factors on IADL to develop specific interventions to improve everyday functioning in persons with mild cognitive disorder. Therefore, this study aimed to 1) explore different influencing factors on IADL functioning considering all domains of the International Classification of Functioning, disability, and health and 2) rank these factors.

**Methods:**

We performed a secondary analysis of a cohort including participants with amnestic mild cognitive impairment (a-MCI) or mild Alzheimer’s Dementia (mild AD). The IADL functioning model was used as a starting point to estimate the effects of cognitive and physical function factors and personal and environmental factors on IADL functioning using multiple linear regression analysis, including subgroup analysis in persons with a-MCI. We used standardized coefficient estimates to relate the size of the predictor effects in the final model.

**Results:**

We included 105 participants (64 a-MCI, 41 mild AD); the mean age was 81.9 years (SD 4.9), with 70% females. Based on a multi-step approach and model fit, the final model included IADL functioning as the response variable and memory, attention, executive function, vision and hearing, mobility, balance, education, and social support as predictors. The final model explained 75% of the variability. The significant predictors in the model were mobility, balance, attention, and education, and were the predictors with the most considerable effects based on standardized coefficient estimates. The subgroup analysis, including only a-MCI participants, revealed a similar pattern.

**Conclusion:**

Our results confirm that IADL functioning in people with mild cognitive disorder is influenced by cognitive and physical function and personal factors. The study provides further insight into understanding IADL functioning impairments in persons with mild impaired cognition and may be used to develop specific non-pharmacological interventions.

**Supplementary Information:**

The online version contains supplementary material available at 10.1186/s12877-022-03476-8.

## Background

Due to demographic changes worldwide, preventing disability caused by neurocognitive impairment is a public health priority [1]. However, it is not fully understood what contributes to disability in persons with neurocognitive impairment and, consequently, how to counteract disability with non-pharmacological interventions. Dementia refers to a family of neurological diseases leading to memory loss and impaired cognitive function, severely enough to affect the performance of everyday activities in daily life. Alzheimer’s disease (AD) is the most common form of dementia and shows different progressive severity stages, e.g., mild, moderate, and severe AD [1]. Mild Cognitive Impairment (MCI), described as the transient state between normal cognitive aging and dementia [2], has widely been used in clinical and research settings to define and study the early stages of dementia [2]. MCI is characterized as no dementia, with a subjective and clinical manifest decline in one or more domains of cognition greater than expected for age with no or only minor impairments in performing instrumental activities of daily living (IADL) [3]. A significant challenge in clinical care is making a clear distinction between MCI and mild AD since overlap occurs between the two conditions [4]. Furthermore, the distinction between cognitively healthy older persons, MCI, and mild AD is also based on how impaired cognition affects daily life activities; however, no clear cut-offs were proposed [5]. Therefore, everyday functioning is an important clinical and diagnostic feature of mild forms of cognitive decline, such as MCI and mild AD [6], and thus worthwhile to investigate more in-depth.

Everyday functioning is – in geriatric literature—generally expressed as basic activities of daily living (BADL) and instrumental activities of daily living (IADL). The latest is crucial to maintain independence in everyday life [7] and includes more complex activities and tasks, e.g., managing finances or doing the shopping [8], while BADL comprise self-care activities, e.g., eating [9], which are mastered early in life, rely mostly on routines and are preserved the longest in the light of cognitive decline when compared to IADLs [10]. Although the advanced (A) ADL, those activities which go beyond independence in daily life (e.g., hobbies, voluntary work), have shown to be most sensitive to early cognitive decline [11, 12], the distinction between healthy persons and people with MCI and between people with MCI and people with AD is more successful with the IADLs [13]. Research on the IADLs, therefore is pivotal. Cumulative evidence illustrates that people with the mild cognitive disorder have minor IADL functioning impairment [14–16]. IADL impairments are relevant to evaluate and manage because they: 1) predate clinical manifest cognitive decline [17] and predict future decline, 2) are associated with reduced wellbeing [18], 3) higher caregiver burden [19], and 4) higher supervision time and total societal costs [20].

IADL functioning is related to an appropriate cognitive function [7] and physical health [21]. Firstly, IADL and cognitive functioning are interrelated [16, 18]. The newly developed Goal-Control-Model provided insight into how cognition affects everyday life activities [19]. However, the model focused on “specific everyday activities (i.e., object-oriented and sequential activities in the service of a practical goal)” [19]. The authors concluded that for these specific everyday activities overall cognition, episodic memory, and executive function are relevant to assessing the level of impairment [19]. However, an earlier Meta-Analysis reported that a large amount of variance in IADL functioning in people with MCI remained unexplained by cognition. In addition, some subdomains of cognition, such as executive functioning, attention, and working memory, were more correlated to IADL than others [22]. This study underlined that other factors than cognition alone might affect IADL functioning [23].

Secondly, literature suggests that people with mild cognitive disorder face difficulties in different aspects of physical functioning. Observational studies reported that people with MCI and mild AD have problems in motor functions [24–26], a higher fall risk [26, 27] and impaired balance [28]. Thirdly, impaired sensory functions have been associated with IADL changes [29]. Seeing and hearing dysfunctions in conjunction with cognitive decline were related to impaired IADL in older people [30]. However, it remains unclear which factors influence IADL functioning and whether some aspects are more important than others. In addition, IADL functioning shows a certain amount of interpersonal variability, as it is associated with various possible influencing factors, e.g., the environment a person lives in or a person’s habits [31]. Moreover, various personal factors (i.e., demographic and clinical characteristics) have been discussed in the literature to influence IADL functioning, such as age [32], education [33, 34], comorbidities [34, 35] and neuropsychiatric symptoms [36].

Thus, IADL are a complex construct, and various factors may play an important role. The complexity of the construct needs to be considered in the design of potentially effective interventions because interventions should be based on a theoretical framework reflecting how an intervention might work [37]. Therefore, it is essential to understand the various factors influencing IADL and how they interact to develop potentially effective interventions for persons at the beginning of a neurocognitive decline.

To determine relevant factors influencing IADL functioning in people with MCI, a model on IADL functioning in people with MCI was developed in a Delphi study [38] using the bio psychosocial model of disability and health, the International Classification of Functioning (ICF) [39] as a model for further discussion. The results suggested that IADL functioning in people with MCI may be associated with cognitive functions, i.e., memory, attention, executive function and executive subdomains reasoning/problem solving and organization/planning; as well as physical functions, i.e., vision, hearing functions, mobility/gait, functional mobility and balance; along with personal, i.e., education and environmental factors, i.e., social network/environment and social support [38]. However, a Delphi study provides only a consensus among experts in a respective field [40].

Therefore, this study aimed to explore empirically 1) whether cognitive and physical function, as well as environmental and personal factors affect IADL functioning in persons with mild impaired cognition, 2) how and to what extent the included factors influence IADL functioning. The results may provide further insight into the relevant factors influencing IADL functioning in mild cognitive disorders. Therefore, it might be used to design specifically targeted non-pharmacological interventions to improve IADL functioning.

## Methods

### Design / database

We performed a secondary analysis of a dataset from a cross-sectional study conducted at the Vrije Universiteit Brussel. The data was consecutively collected between November 2014 and March 2018 in a geriatric day hospital of an academic teaching hospital (UZ Brussel, Belgium) to develop an assessment tool to evaluate everyday functioning in neurocognitive disorders [41, 42]. All patients were asked if they wanted to participate in the study and if so, they signed an informed consent form. The database included a well-defined cohort of elderly people (*N* = 114), from participants with MCI (*n* = 65) or mild AD (*n* = 49), encompassing several measurements on functional performance, global cognitive function, cognitive domains, physical functions, depression, and medical history.

### Participants

The cohort included community-dwelling persons, > 70 years of age with stable medical conditions, referred to the geriatric day hospital for a cognitive diagnostic workout and accompanied by a partner/caregiver (spouse, family, or close friends) who could provide independent and accurate information about the persons’ functional status [10]. The exclusion criteria of the original study were: taking antidementia drugs, having sensory or communicative impairments that preclude participating in the assessment procedure, history of major psychiatric illness or other neurological diseases than a-MCI or mild AD (e.g., Parkinson’s Disease, stroke, or epilepsy) [10].

Participants underwent a standardized multidisciplinary clinical assessment procedure [43]. The process included medical history taking and extensive neuropsychological assessment, neurological and physical examination, functional evaluation, extensive laboratory blood testing and brain imaging by CT or MRI scan. In an interdisciplinary team and based on the results from the multidisciplinary diagnostic procedure, participants were diagnosed with amnestic (a-)MCI, fulfilling the diagnostic criteria of a-MCI [44] or mild AD, fulfilling the National Institute of Neurological and Communicative Disorders and Stroke – Alzheimer’s Disease and Related Disorder Association (NINDS-ADRDA) [45].

For the secondary analysis, we only included participants with a diagnosis of a-MCI or mild AD. Additionally, participants were excluded from the analysis when low scores on cognitive tests could point to a moderate cognitive impairment through their clinical diagnosis, in order to obtain a clear sample. Therefore, persons with scores less than 19/30 in the Mini-Mental State Examination (MMSE) [46] or < 50/105 in the Cambridge Cognitive Test-Revised (CAMCOG-R) [47] were extra excluded. We also only analyzed complete cases.

### Measurements

IADL functioning was assessed using the Instrumental Activities of Daily Living scale as part of the Brussels Integrated Activities of Daily Living Inventory (BIA) [13]. The BIA and particular of interest for this paper, the i –ADL tool has shown to be reliable and valid in identifying cognitive disorders in a geriatric population [13, 41, 42]. The tool evaluates the nine IADLs from the LAWTON scale [48] by informant report. Informants were relatives or caregivers (spouse, family, or close friend), interacting with the person close enough to provide accurate information about IADL performance. The tool considers only the relevant activities. Relevant activities were activities that the participant currently or lately performed since the years of retirement. This is important because, some of the activities may have never been performed before and are therefore irrelevant to evaluate for the participant. The total number of relevant activities was calculated [10]. Next, the informant was asked how the relevant activities were currently performed and the interviewer assigned a score ranging from 0 (no difficulty to perform) to 4 (complete difficulty to perform), based on the performance qualifiers of the ICF [39]. Each activity with a score of more than zero was counted as an activity with limitations. Based on this information, a total i-ADL disability index (i-ADL-DI) was calculated, considering the sum of relevant activities, the number of limited activities, and each limitation’s severity [41]. The i-ADL-DI was expressed as a percentage with higher scores representing worse functioning.

Memory was assessed using two measurement scales: 1) the memory subscale from the Alzheimer’s Disease Assessment Scale, cognitive subscale (ADAS-cog) [49], score range from 0 to 30, and a higher score indicate better functioning; 2) the memory subtest from the CAMCOG-R, the score ranges from 0 to 27; higher score indicates better functioning [47].

Attention was assessed using: 1) the attention/calculation subscale from the CAMCOG-R, the score ranges from 0 to 9; higher score indicate better functioning [50]; or 2) the Trail Making Test, part A (TMT-A) [51], the time needed to complete the task was measured in seconds; higher scores indicate worse functioning.

Executive function was assessed with 1) the Frontal Assessment Battery (FAB) [52], score range 0 to 18, and a higher score indicates better functioning; 2) the Trail Making Test part B (TMT-B) [51], the time needed to complete the task was recorded in seconds; higher scores indicate worse functioning. We did not use the generally used cut-off of 300 s in the recordings of TMT-A and TMT-B [53], instead the time a person needed to fulfill the task was used.

Sensory functions (i.e., vision and hearing) were based on medical history. The presence of an impairment of vision or hearing was determined if the participant stated as having difficulties with his sensory functions, regardless of using an aid. The scores are dichotomized (i.e., yes or no).

Mobility was assessed by the four-meter walking test [54]. The test measures gait speed in seconds over four meters [55]. The mean of two subsequent recorded cycles was calculated, while higher scores indicate worse functioning.

Balance was assessed by the Tinetti Test [56, 57]. The test includes static, dynamic, reactive, and anticipatory balance measures and measures of ambulation and transfer ability (score range 0 to 28; higher scores indicate better functioning).

Education was assigned in accordance with the Belgium education system and were transformed into years in education, ranging from 6 to 17 years.

Social network/support was operationalized whether a person was living alone or not.

### Variable selection

A priori, we selected the variables to be included in the multivariate analysis based on a multi-step approach [58]. First, we determined the variables that best operationalized the response variable (IADL functioning) and factors from the IADL functioning model (predictors) based on the literature [4]. Second, the variables were explored on missing values. Third, the continuous variables were examined on their dispersion (range) to ascertain that they have a certain amount of variance [58].

### Data analysis

All analyses were performed using the R statistical software R version 4.2.0 [59]. The complete R Script of the data analysis is included in the Additional file 1. An alpha level of 0.05 was required for statistical testing as otherwise indicated.

The data was explored through descriptive summaries to detect the proportion of missing values and determine the variability. In addition, distributions and dispersions of the response variable and predictors were visually inspected. We investigated inter-variable correlations using all complete pairs of observations on respective variables (Pearson, respectively point-biserial, if appropriate). Due to the natural similarity between some predictors (e.g., mobility and balance, cognitive domains), we determined a too high inter-variable correlation of more than 0.9 [60]. Pairwise scatterplots were established of all continuous variables to explore a linear relationship between the response variable and predictors.

We fitted multiple linear regression models to the complete cases using the lm function from the base package within R. IADL functioning was the response variable, and attention, memory, executive function, vision, hearing functions, mobility, balance, social support, and education were predictors. We defined a priori to select the model based on the Akaike Information Criterion (AIC) [61] when more than one variable was available for a particular predictor of the IADL functioning model (i.e., memory, attention and executive function).

Model diagnostics included investigation of 1) heteroscedasticity by visual inspection of Tukey-Anscombe plots and using the studentized Breusch-Pagan Test, 2) multicollinearity through the Variance Inflation Factor (VIF), and 3) we used Cook’s Distance (Cook’s D) to detect unusual data points [58]. A general guideline is that $\sqrt{\mathrm{VIF}}$ > 2 would be a sign for multicollinearity and a Cook’s D > 4/(*n*-*k*-1), where *n* is the sample size and *k* the number of predictors, would indicate unusual data points [58]. We reran the analysis using the z-scores of the response variable and predictors. The standardized coefficient estimates were plotted to visualize the corresponding effects and 95% confidence intervals.

In addition, we performed a subgroup analysis including only the participants with an a-MCI diagnosis because several demographic and clinical characteristics differed substantially between the two diagnostic groups, and because the IADL functioning model was developed for individuals with MCI [38]. We fitted a multiple linear regression model including the same response variable and predictors. The model fit was investigated using the visual inspection of residuals, studentized Breusch-Pegan Test, VIF and Cook’s D.

## Results

We additionally excluded one participant with a-MCI and eight participants with mild AD from the dataset, because they were subjective to moderate cognitive decline based on their scores on the MMSE and CAMCOG-R. Therefore, we included the data from 105 participants in the analysis, 64 (61%) of the participants were diagnosed with a-MCI, and 41 (39%) were diagnosed with mild AD. Table 1 provides the demographic and clinical characteristics of all participants. The two groups were comparable considering demographic characteristics of age, sex, education, number of children and number of comorbidities. People diagnosed with a-MCI had a higher level of cognitive functioning based on the MMSE and the CAMCOG-R total score.

**Table 1:** Demographic and Clinical Characteristics of Participants

	Participants (*n* = 105)				
		a-MCI (*n* = 64)	mild AD (*n* = 41)	Test statistic^a^	*p*-value
Age, years					
Mean (SD) Range	81.9 (4.9) 71 – 96	81.7 (4.9) 71 – 96	82.3 (4.9) 74 – 92	t(86) = -0.65	*p* = .52
Sex, female					
Frequencies (%)	74 (70%)	44 (69%)	30 (73%)	*Χ*^*2*^ = 0.23	*p* = .67
Education, years					
Mean (SD) Range	12.9 (1.8) 6 – 17	13.1 (1.9) 6 – 17	12.9 (1.8) 6 – 17	t(89) = 0.58	*p* = .57
Children					
Median (IQR) Range	2 (2) 0—9	2 (2) 0—9	2 (2) 0—6	t(93) = -0.78	*p* = .43
Comorbidities					
Median (IQR) Range	5 (3) 1 – 13	5 (3) 1 – 13	5.5 (3) 2 – 12	t(87) = -0.67	*p* = .51
MMSE					
Mean (SD) Range	24.4 (2.8) 19 – 30	25.6 (2.3) 20 – 30	22.5 (2.3) 19 – 27	t(87) = 6.70	*p* < .001
CAMCOG total					
Mean (SD) Range	78.9 (7.9) 50 – 95	82.7 (5.6) 72 – 95	73.3 (7.8) 50 – 87	t(67) = 6.75	*p* < .001

*SD* Standard Deviation, *IQR* Interquartile Range, *MMSE* Mini Mental State Examination, *CAMCOG* Cambridge Cognitive Test, *a-MCI* Amnestic mild cognitive impairment, *Mild AD* Mild Alzheimer’s Disease

^a^Differences between groups, MCI versus Mild AD; Welch two-sample t-test, Chi-square test if appropriate

**Fig. 1:**
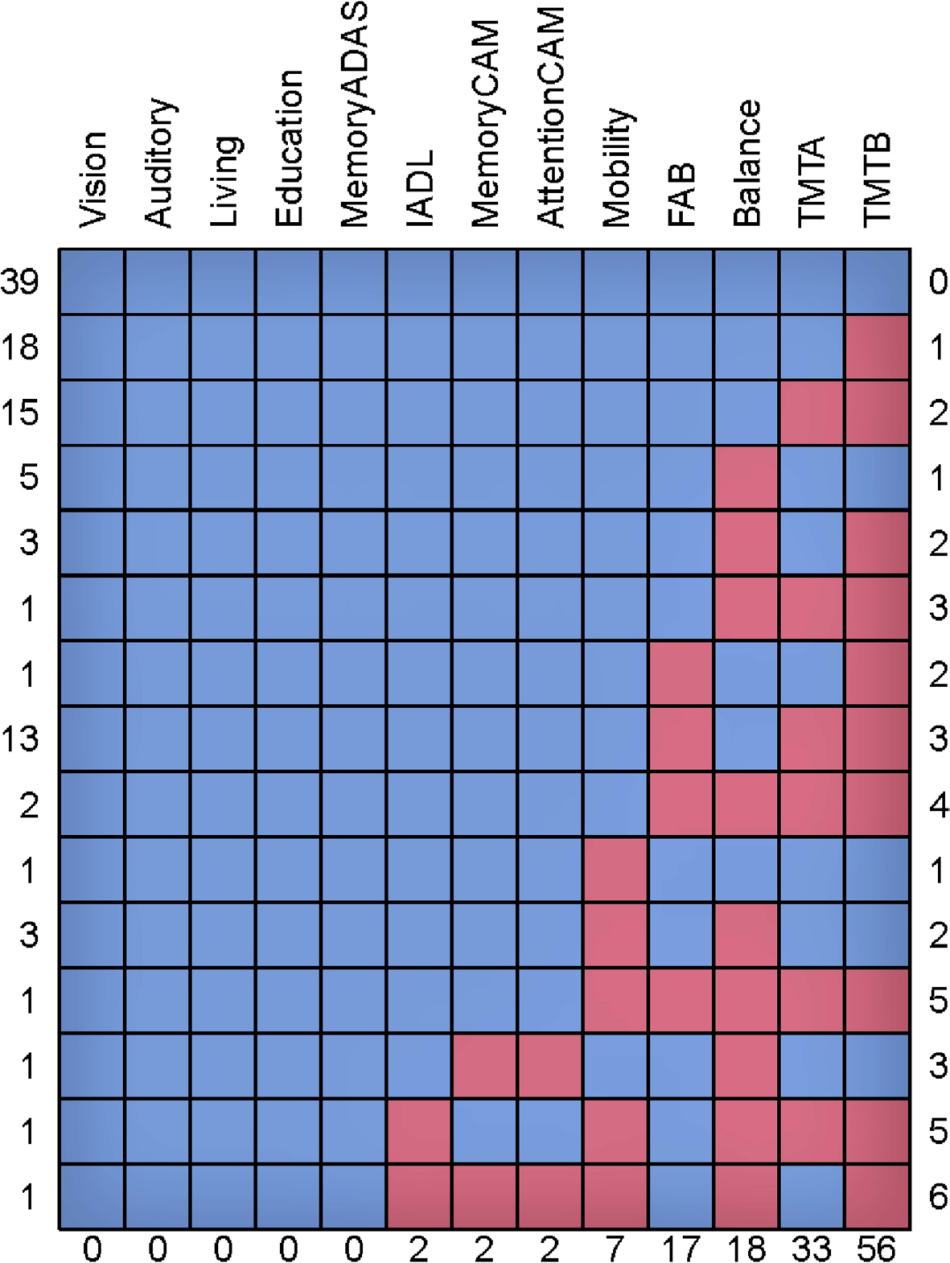
Missing data pattern, including all participants (*n* = 105). Detailed legend: Each row corresponds to a missing data pattern, 1 = observed and 0 = missing. Rows and columns are sorted in increasing amounts of missing information. The last column and row contain row and column counts, respectively. Abbreviations: memoryadas, Memory Subscale Alzheimer’s Disease Assessment Scale; IADL, Instrumental Activities of Daily Living; memoryCAM, Memory Subscale Cambridge Cognitive Test-Revised; attentionCAM, attention subscale Cambridge Cognitive Test-Revised; FAB, Frontal Assessment Battery; TMTA, Trail Making Test part A; TMTB, Trail Making Test part B

Based on the multi-step approach, the following predictors were selected: memory, attention, executive function, mobility, balance, social support, and education. The visual inspection of distributions and dispersions of the response variable and predictors gave no indication of multimodal distributions or too small variability. Pairwise inter-variable correlations ranged from -0.002 (vision and memory measured using the CAMCOG-R subscale) to -0.069 (balance and mobility); all inter-variable correlations can be found included in the Additional file 2. Pairwise scatterplots indicated linearity between the response variable and predictors (data not shown). Figure 1 provides an overview of the missing data pattern. Four predictors had more than 15% of missing data (i.e., balance, executive function measured using the TMT-B, executive function measured using the FAB and attention measured using the TMT-A).

Table 2 provides all clinical measures of the response variable (i.e., IADL functioning using the i-IADL-DI) and the predictors. The following variables differed between participants with a-MCI and mild AD: IADL functioning; memory, measured using ADAS-cog subscale and CAMCOG-R subscale; attention measured using TMT-A; and executive function, measured using FAB and TMT-B.

**Table 2:** Clinical measures of the dependent variable and independent variables

	Participants			
		a-MCI	mild AD	Test tatistic^a^ *p*-value
IADL (i-ADL DI, %)				
Mean (SD) Range	41 (22) .0 – 94 (*n* = 103)	35 (23) .0 – 86 (*n* = 62)	50 (18) 6 – 94 (*n* = 41)	t(98) = -3.75 *p* < 0.001
Memory^b^ (ADAS-cog;./30)				
Mean (SD) Range	12.5 (3.6) 4 – 22 (*n* = 105)	13.7 (3.5) 4 – 22 (*n* = 64)	10.6 (3.0) 5 – 18 (*n* = 41)	t(93) = 4.84 *p* < 0.001
Memory^c^ (CAMCOG-R;./27				
Mean (SD) Range	15.5 (3.9) 4 – 27 (*n* = 103)	17.1 (3.2) 10 – 27 (*n* = 62)	13.1 (3.4) 4 – 20 (*n* = 41)	t(82) = 5.93 *p* < 0.001
Attention (TMT-A, s)				
Mean (SD) Range	72.9 (46.9) 38.0 – 240.0 (*n* = 72)	67.6 (21.0) 38.0 – 119.9 (*n* = 44)	123.8 (55.5) 51.5 – 240.0 (*n* = 28)	t(32) = -4.97 *p* < 0.001
Attention^d^ (CAMCOG-R,./9)				
Mean (SD) Range	7.0 (1.8) 2 – 9 (*n* = 103)	7.2 (1.7) 2 – 9 (*n* = 62)	6.7 (1.7) 3 – 9 (*n* = 41)	t(86) = 1.56 *p* = 0.12
Executive function (FAB,./18)				
Mean (SD) Range	13.24 (2.9) 5 – 18 (*n* = 88)	13.8 (2.6) 8 – 18 (*n* = 55)	12.3 (3.4) 5 – 18 (*n* = 33)	t(54) = 2.12 *p* < 0.05
Executive function (TMT-B, s)				
Mean (SD) Range	218.2 (108.6) 50 – 536 (*n* = 49)	194.9 (102.1) 50 – 536 (*n* = 38)	298.8 (93.6) 158 – 450 (*n* = 11)	t(18) = -3.18 *p* < 0.01
Mobility (4-m test, s)				
Mean (SD) Range	5.4 (2.2) 3 – 15.1 (*n* = 98)	5.4 (2.1) 3 – 12.4 (*n* = 58)	5.5 (2.5) 3.3 – 15.1 (*n* = 40)	t(74) = -0.31 *p* = 0.75
Balance (Tinetti,./28)				
Mean (SD) Range	24.5 (2.9) 7 – 28 (*n* = 87)	24.6 (4.4) 7 – 28 (*n* = 52)	24.3 (4.7) 9 – 28 (*n* = 35)	t(69) = 0.29 *p* = 0.77
Education (years)				
Mean (SD) Range	13.0 (1.8) 6 – 17 (*n* = 105)	13.1 (1.9) 6 – 17 (*n* = 64)	12.9 (1.8) 6 – 17 (*n* = 41)	t(89) = 0.58 *p* = .57
Vision (impaired)				
Frequencies (Percent)	20 (19%) (*n* = 105)	14 (22%) (*n* = 64)	6 (15%) (*n* = 41)	*Χ*^*2*^ = 0.85 *p* = .43
Hearing (impaired)				
Frequencies (Percent)	11 (10%) (*n* = 105)	8 (12.5%) (*n* = 64)	2 (5%) (*n* = 41)	*Χ*^*2*^ = 2.25 *p* = .19
Living (alone)				
Frequencies (Percent)	55 (52%) (*n* = 105)	36 (56%) (*n* = 64)	19 (46%) (*n* = 41)	*Χ*^*2*^ = 0.98 *p* = .43

*IADL* Instrumental Activities of Daily Living, *i-ADL DI* Instrumental Activities of Daily Living Disability Index, *CAMCOG-R* Cambridge Cognitive Test-Revised, *ADAS-cog* Alzheimer’s Disease Assessment Scale, cognitive subscale, *TMT—A* Trail Making Test part A, *FAB* Frontal Assessment Battery, *TMT-B* Trail Making Test part B

^a^Differences between groups, MCI versus mild AD; Welch two-sample t-test, Chi-square test, if appropriate, ^b^Memory subscale ADAS-cog, ^c^Memory subscale CAMCOG-R, ^d^Attention/Calculation subscale CAMCOG-R

### Multivariate analysis

We fitted eight linear regression models. The final model was selected based on the smallest AIC. The final model included IADL function as the response variable and memory based on the ADAS-cog subscale, attention based on the TMT-A, and executive function using the TMT-B, mobility, balance, social support, and education as predictors. The residual standard deviation was 0.14 on 29 DF, the *R*^*2*^ = 0.75 and F-statistic 9.807 on 9 and 29 DF (*p* < 0.001). Table 3 provides the coefficient estimates of the predictors based on the linear regression analysis. Mobility, balance, education, and attention were significant predictor variables in the model. Overall, the standard errors were substantial compared to the coefficient estimates. Visual inspection of Pearson residuals indicated no evidence of a violation of model assumptions. The studentized Breusch-Pagan test did not reject homoscedasticity (BP(9) = 4.48, (*p* = 0.877)). Cook’s D values were in the range of 0.22 to 0.000005, and the results from the VIF test indicated no sign of multicollinearity. The Cook’s D and VIF test results can be found in Additional file 3.

**Table 3:** Coefficient estimates and standardized coefficient estimates including participants with a-MCI and mild AD

Predictor	Coefficient Estimate	Standard Error	95% CI	Standardised Estimate	95% CI	t-statistic	
Intercept	-0.01	0.43	[ -0.90, 0.87]	< 0.01	[-0.19, 0.19]	-0.03	
Vision (impaired)	-0.13	0.10	[ -0.33, 0.07]	-0.16	[-0.41, 0.09]	-1.30	
Hearing (impaired)	0.32	0.18	[ -0.04, 0.69]	0.21	[-0.03, 0.45]	1.81	
Mobility	0.08	0.02	[ 0.04, 0.12]	0.52	[ 0.28, 0.77]	4.37	^***^
Balance	-0.02	0.01	[ -0.04, > -0.01]	-0.29	[-0.53, -0.05]	-2.51	^*^
Living (together)	0.02	0.05	[ -0.08, 0.12]	0.04	[-0.16, 0.25]	0.45	
Education	0.03	0.01	[ 0.00, 0.06]	0.23	[ 0.02, 0.45]	2.21	^*^
Memory	-0.01	0.01	[ -0.03, 0.01]	-0.14	[-0.37, 0.09]	1.21	
Attention	< 0.01	< 0.01	[< 0.01, < 0.01]	0.27	[ 0.03, 0.52]	2.31	^*^
Executive Function	< 0.01	< 0.01	[> -0.01, < 0.01]	0.17	[-0.09, 0.44]	1.33	

*CI* Confidence Interval

Significance levels: ^*^p < 0.05; ^***^, *p* < 0.001

**Fig. 2:**
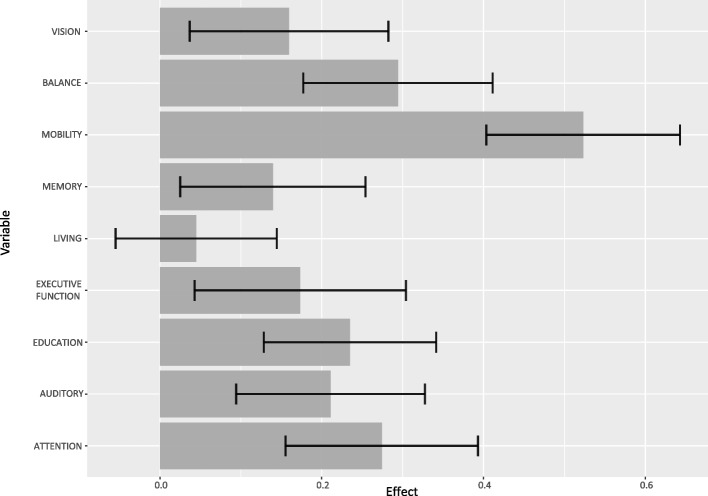
Standardized coefficient estimates of the linear regression model including participants with a-MCI and mild AD. Detailed legend: Dark grey bars show the standardized coefficient estimates of the predictor variables from the linear regression model, the black lines the corresponding standard error

Figure 2 provides a graph of the standardized coefficient estimates of the predictor variables, including 95% confidence intervals. Ranking the predictors based on their standardized coefficient estimates (Table 3) indicated mobility, balance, attention, education as the predictors with the most considerable effects.

Subgroup analysis using the final linear regression model including participants with a-MCI had the following results: the residual standard deviation was 0.16 on 19 DF, the *R*^*2*^ = 0.77, and F-statistic 7.129 on 9 and 19 DF (*p* < 0.001). Table 4 provides the coefficient estimates of the predictors based on the linear regression analysis. Mobility, balance and education were significant predictor variables in the model.

**Table 4:** Linear regression predictor coefficient estimates including participants with a-MCI

Predictor	Coefficient Estimate	Standard Error	95% CI	t-statistic
Intercept	0.11	0.60	[ -1.15, 1.37]	0.19
Vision (impaired)	-0.24	0.14	[ -0.54, 0.05]	-1.73
Hearing (impaired)	0.41	0.21	[ -0.03, 0.85]	1.94
Mobility	0.07	0.02	[ 0.03, 0.12]	3.39^**^
Balance	-0.03	0.01	[ -0.06, > -0.01]	-2.49^*^
Living (together)	-0.02	0.06	[ -0.15, 0.12]	-0.26
Education	0.04	0.02	[< 0.01, 0.08]	2.10^*^
Memory	-0.01	0.01	[ -0.03, 0.01]	0.99
Attention	< 0.01	< 0.01	[> -0.01, 0.01]	1.45
Executive Function	< 0.01	< 0.01	[> -0.01, < 0.01]	1.66

*CI* Confidence Interval

Significance levels: ^*^p < 0.05 ^**^, *p* < 0.01

Model diagnostics indicated no sign of model assumption violations, i.e., residual analysis, heteroscedasticity, multicollinearity, and unusual data points. The results on the model diagnostics can be found in Additional file 3.

## Discussion

We aimed to explore whether, how, and to what extent predictors covering all different domains of the ICF affected IADL functioning in persons with mild impaired cognition. As far as we know, this was the first study that empirically investigated the influence of different aspects of human functioning, i.e., cognitive and physical functions, personal and environmental factors, on IADL functioning in the same sample based on a theoretical model [38].

The results from our multivariate analysis indicated that our model explains 75% of variability in IADL functioning with mobility, balance, education, and attention as significant predictors with – compared to the other predictors—the most considerable effect. In the subgroup of persons with a-MCI we observed a similar pattern, except attention was no longer a significant predictor.

Primarily cognitive function has been discussed in the literature among the different domains of the ICF that may affect IADL functioning in persons with mild impaired cognition. This is not surprising, as the definition of IADL describes IADL as activities that require higher-order cognitive processes [62]. For example, an observational study found in persons with MCI that a high cognitive demanding factor from the Bayer-Activities of Daily Living Scale was associated with the cognitive domains of memory, attention/processing speed, executive function, language, and visuospatial function [63]. Overall, the standardized effects of the three cognitive domains, attention, memory, and executive function, were not the most prominent predictors, aligning with the literature. McAlister et al. [22] investigated the association between cognitive domains and functional abilities in people with mild impaired cognition in their meta-analysis. Overall, cognition accounted for only 23% of the variability in IADL functioning, whereas the authors concluded that "a large amount of variance remained unexplained by cognition" [22]. Our study results indicated attention to be the only significant predictor in the model and the predictor with the third-largest effect. This finding suggests that persons facing problems to direct and maintain their attention may have more functional problems, which is in line with the literature. In a meta-analysis among the cognitive domains, attention accounted for 33% of the variability in IADL functioning [22].

Memory and executive function were no significant predictors and ranked lower than the others (sixth, eighth, respectively). This was unexpected because memory impairment is one of the main symptoms observed in people with mild impaired cognition [5]. A previous study using the i-ADL-DI of the Brussels Integrated Activities of Daily Living Inventory (BIA) do describe executive functions as contributor to everyday functioning in IADL [10]. Furthermore, this finding contradicts the literature [22, 63]. Recently, the integrated Goal-Control-Model was proposed to explain the cognitive processes necessary for functioning in everyday activities to advance the assessment of everyday activities and develop targeted interventions to improve these activities [64]. However, the Goal-Control-Model is narrowed down to specific everyday activities and excludes relevant IADLs such as communication, transportation and managing finances [64]. Nonetheless, the authors suggest that overall cognitive function, episodic memory, and executive function are relevant to assessing functional impairment in persons with mild impaired cognition.

The standardized coefficient estimates of executive function in our model indicated that the impact of executive function might be limited, in contrast to the literature. Other studies found that executive function impacts IADL functioning in people with mild impaired cognition [10, 65, 66]. The results of our multivariate analysis may be contributed by the number of missing values in the TMT-B, indicating that the heterogeneity of the predictor was limited. One explanation for the high number of missing values is that the test was too difficult for these participants. In addition, executive functions are a broadly defined umbrella term used for higher-order cognitive processes that coordinate cognitive, emotional and motor activity during the performance of BADLs and IADLs [67]. Executive functions organize behavior, generate, or inhibit responses and may include the subdomains of planning, organization, decision-making, working memory, responding to feedback, inhibition and flexibility [5]. The divergent findings may represent the heterogeneous definitions of executive function, subdomains, and operationalization. Therefore, the impact of executive function might still be relevant, even if our study did not confirm this.

Literature suggests a possible association of additional cognitive domains on IADL functioning in people with mild neurocognitive disorder. Results from a cohort study indicated that language might be associated with IADL functioning in people with MCI [66]. Because language was not included in the IADL functioning model and our analysis was theory-driven, we did not include language as a predictor in the model. Another study reported that intelligence accounts for 50% of the variability in IADL functioning in people with mild neurocognitive disorder [68]. One might argue that intelligence is a personal factor and not a cognitive function. Based on the ICF, intelligence is assigned to cognitive functions (b117) [39]. Despite the existence of specific linking rules, linking e.g., an item in a measurement tool or an intervention aspect to a certain component of the ICF is not always straightforward [69]. In addition, personal factors are currently recorded but not classified in the ICF [39]. It is possible that the predictors presented in our study differ from the literature due to our methodological approach. We did not conduct an exploratory analysis, but a theory-driven approach [58]. Nevertheless, for future studies, we would recommend including language, intelligence, and other domains of cognition, such as working memory, because the results of our study indicated the model might be not comprehensive.

As for physical function, mobility – operationalized with the 4-m walking test—was a significant predictor in our model and the factor with the largest effect based on the standardized coefficient estimate, which was in line with the literature [25, 70–72]. For example, a meta-analysis reported that a low gait speed resulted in reduced IADL function [25]. In the present study, the mean value for gait speed would not indicate a severely impaired sample in this age group. However, the ranges were wide, with a minimum value of 0.26 m/s, which would be highly susceptible to impaired mobility. The results from the multiple linear regression analysis can be interpreted that a one-point change in mobility would correspond to a 0.08 (8% on the iADL-DI) decrease in IADL function controlled for all other predictors. However, the standard errors were substantial. In addition, as our analysis was explorative, our results may not be valid for other samples. Nevertheless, one might conclude that mobility is an important influencing factor on IADL functioning in people with mild cognitive disorder. However, further studies are needed to investigate the association of cognitive decline, mobility impairment and IADL functioning limitations at early stages of cognitive decline. Furthermore, although it seems reasonable that gait speed influences IADL functioning in elderly people with mild impaired cognition it remains unclear, whether an improvement in gait speed or mobility due to an intervention would result in better IADL functioning.

Our results indicated balance, measured using the Tinetti test, as a significant predictor in the model, and the predictor with the second largest standardized coefficient estimate; which was also in line with the literature [73]. The mean score in our study was not susceptible for an impaired sample, however, the lowest score was 7/28. The observed coefficient estimate for balance, can be interpreted that a one-point change in balance would correspond to a 0.02 (2% on the iADL-DI) increase in IADL functioning, controlled for all other predictors. However, this result should be interpreted with caution due to large standard errors and the size of the effect. It is not surprising that mobility and balance show a similar pattern in our model, because mobility and balance are related constructs, i.e., mobility requires an appropriate level of dynamic balance. Furthermore, the two predictors showed the highest inter-correlation. Functional mobility includes the concept of mobility, static and dynamic balance. Therefore, it could be interesting to investigate functional mobility alone as an influencing factor, e.g., by using the Modified Physical Performance Test [74]. Furthermore, functional mobility was included in the IADL functioning model as a relevant influencing factor of IADL functioning in persons with MCI [38].

Concerning sensory functions, the results from our linear regression model showed that auditory and seeing functions showed a moderate standardized coefficient estimate in relation to the other predictors. However, the predictors were not significant, in contrast to the literature, that suggests that sensory functions, and seeing and hearing functions in particular, might affect IADL functioning in older adults with and without mild cognitive disorder [29, 30]. The contradictory finding may result from the operationalization of the two predictors, because they relied on the participants self-report or informant-report whether an impairment is present or not and did not consider a measured ability to see or hear. Therefore, the role and possible impact of sensory functions on IADL functioning in persons with mild impaired cognition needs to be further investigated.

Education was a significant predictor in our linear regression model, with the third largest effect based on the standardized coefficient estimate. It is well known, that cognitive function in the aged is associated with education as it is seen as a protective factor for cognitive decline and dementia [75]. However, the literature is inconclusive whether education also affects IADL functioning. While a meta-analysis concluded that education was not a mediator of the association between cognitive function and IADL function [29], other studies suggested that a lower level of education was associated with IADL impairments [33, 34]. Our results indicate that education might influence, at least to some extent, IADL functioning in people with mild cognitive disorder.

We included social network/environment operationalized whether a participant was living alone or not as a proxy measure for having support. The predictor was not significant and had a minor effect based on the standardized coefficient estimate related to the other predictors. Nevertheless, the confidence interval was substantial, indicating that the living situation may also have a negative effect on IADL functioning. Based on these results, one could speculate that living together with a caring relative would reduce the capability of functioning. However, it is possible that deviating scenarios occur in real life. On the one hand that there will be no care need because the caregiver resolves the problems in everyday activities; on the other hand, presence of a relative prevents the person with a cognitive impairment from solving everyday problems themselves. Notwithstanding, reduced autonomy in everyday activities are associated with higher caregiver burden, reduced quality of life [19], and higher supervision time [20]. Besides, it should be considered that social network/environment is only partially represented in the living situation. Therefore, it will be essential to investigate the impact of social network/environment and the support provided by the social network/environment in future studies. Ideally, the different aspects of network/social environment and support will be operationalized by more sophisticated assessment tools such as the Inventory of Socially Supportive Behaviors, the Social Provision Scale or the ENRICHD Social Support Inventory [76].

IADL are a complex construct. Therefore, non-pharmacological interventions targeting IADL functioning to sustain independence in persons with mild impaired cognition require multicomponent interventions targeting various domains of the ICF. Designing complex interventions imply a solid theoretical background on the why and how an intervention might work [37]. Motamed-Jahromi & Kaveh proposed a logical model based on a systematic review on effective interventions to improve IADL to be used as a basis for designing new multi-component interventions targeting IADL in persons with MCI [77]. The proposed theoretical framework included a situation analysis, including cognitive and physical function and the personal environment. However, the theoretical framework provided no guidance on which aspects of cognitive and physical function should be examined [77]. Based on our results it seems that multi-component interventions should at least include a component to train physical fitness aspects to improve mobility (e.g., balance, endurance, strength, and flexibility); and a cognitive training component to improve attention. Recently, a review proposed to consider theoretically motivated language interventions in the design of interventions targeting everyday functioning, because "cognitive models of language production and everyday action share a number of similarities" [78]. However, this review focuses on the cognitive aspects of everyday functioning. Therefore, our study may support the understanding of correlates of IADL functioning in persons with mild impaired cognition and thus, drive the development of specifically designed non-pharmacological multicomponent interventions.

### Limitations

We reported on a secondary database analysis; therefore, the data was not primarily collected for our study. Therefore, our research and the results relied on the variables included in the database. However, the original study and set of variables also followed the ICF model [41, 42], and therefore, was suitable for our study. Furthermore, the results should be interpreted considering the operationalization of the predictors and response variable, the selection of variables and our data analysis approach. We abandoned analyzing the data through an explorative process, e.g., stepwise backward selection of variables. Instead, we used a theory-driven approach using the IADL functioning model to avoid biases in our results. Thus, we did not explore other potentially influencing factors, e.g., language, and as such, the model might be further developed in future studies.

As for our response variable, IADL functioning was operationalized using the i-IADL disability index. The i-IADL DI relies on informant reported problems in performing the relevant activities. There is an ongoing debate on the optimal modality of assessment of everyday functioning, i.e., performance-based assessments versus self- or informant-reported questionnaires, because no gold standard exists [7, 16]. It is argued that performance-based assessment tools capture different aspects of everyday functioning than informant-reported questionnaires [79]. Although the i-IADL DI has a robust convergent validity with the Naturalistic Action Test [80] and discriminates similar between healthy older adults and persons with mild cognitive disorder [42] we cannot rule out the assessment modality may have influenced our results.

There is a vast number of possible activities that can be designated as IADL, and depending on the type of activity, it is conceivable that different predictors could have a varying degree of influence on the performance of the specific activity. The i-IADL DI considers the activities from the nine activities of the LAWTON scale that were relevant to the participants. What activities were finally considered in the i-IADL DI was not further investigated. Therefore, we cannot rule out the possibility that the interpretation and transfer of our results to other activities that are not considered in the LAWTON scale might be limited. However, the LAWTON scale has been a widely used tool in clinical practice for years to assess IADL performance. In addition, the development of the BIA was based on the ICF, so we believe that our results are valuable to understanding the possible factors influencing IADL performance in people with aMCI and mild AD.

The sample size used for the multiple linear regression analysis may have influenced our results. In addition, several predictors had more than 15% missing data resulting in only 47 complete cases in the linear regression model. The high number of predictors in our model in relation to the small number of complete cases may have influenced our results and are reflected in the wide confidence intervals of coefficient estimates. We did not use multiple imputation methods to account for the high number of missing values because imputations rely solely on the observed data and would therefore reduce heterogeneity. Thus, it remains unclear whether our results could be reproduced in another sample. However, we reran the linear regression model in the subgroup of individuals with a-MCI and observed similar results, and therefore, increase our confidence in our results. Our study may serve as a first attempt to better understand the influencing factors on IADL functioning, nonetheless, further studies are needed.

The a-priori defined multi-step approach of predictor selection may have influenced our results. For instance, the predictor memory was measured based on different assessment tools, ADAS-Cog and CAMCOG-R memory subscale. Therefore, selecting other variables as predictors may have changed our results. However, all the mentioned assessment tools have good psychometric properties, are well known and broadly used in memory clinics, representing the daily clinical work in memory clinics, suggesting that our study used a clinically relevant method.

All included participants were older than 70 years, and the mean age was above 80 years. Higher age is a risk factor for cognitive disorders and other diseases and comorbidities, influencing daily functioning [14, 81]. We did not include age as a predictor in our multiple linear regression model because we used a theory-driven approach to select the predictors. Therefore, we cannot rule out, that age may have influenced our results. Hence, our results may not be valid for younger individuals with mild cognitive impairment. Furthermore, IADL functioning may be more strongly influenced by this cohort’s physical functioning as ageing may negatively impact physical functioning [82] and may explain the prominent physical function predictors. However, cumulative evidence suggests that physical functioning is an important risk factor for functional impairment regardless of advanced age [83].

## Conclusion

In conclusion, our results indicated that IADL functioning in people with mild impaired cognition is influenced by cognitive and physical functioning factors and personal factors. Therefore, this study may serve as ground for further exploring IADL functioning in people with a mild impaired cognition such as a-MCI and mild AD and as a basis to develop specific targeted non-pharmacological interventions to improve everyday activities.

## Supplementary Information

**Additional file 1.**

**Additional file 2: Table 1.** Pairwise Inter-variable correlations.

**Additional file 3: Table 2.** Cook’s Distance (Cook’s D) of the model including complete cases of participants with aMCI and mild dementia. **Table 3.** Variance Inflation Factor (VIF) coefficient estimates of the predictors including complete cases of participants with aMCI and mild dementia. **Table 4.** Cook’s Distance (Cook’s D) of the model including complete cases of participants with a-MCI. **Table 5.** Results of studentized Breusch-Pagan test model including complete cases of participants with a-MCI. **Table 6.** Variance Inflation Factor (VIF) coefficient estimates of the predictors including complete cases of participants with a-MCI. **Figure 1.** Residual versus Fitted values, model including complete cases of participants with aMCI and mild dementia. **Figure 2.** Normal Q-Q Plot, model including complete cases of participants with aMCI and mild AD. **Figure 3.** Scale Location, model including complete cases of participants with aMCI and mild AD. **Figure 4.** Residual versus Fitted values, model including complete cases of participants with a-MCI. **Figure 5.** Normal Q-Q Plot, model including complete cases of participants with a-MCI. **Figure 6.** Scale Location, model including complete cases of participants with a-MCI.

## Data Availability

All data used in this study are available on request from the Vrije Universiteit Brussels.

## Abbreviations

AD: Alzheimer’s Dementia
ADAS-cog: Alzheimer’s Disease Assessment Scale, cognitive subscale
AIC: Akaike Information Criterion
BADL: Basic Activities of Daily Living
BIA: Brussels Integrated Activities of Daily Living Inventory
CAMCOG-R: Cambridge Cognitive Test-Revised
CI: Confidence Interval
Cook’s D: Cook’s Distance
DF: Degrees of Freedom
FAB: Frontal Assessment Battery
IADL: Instrumental Activities of Daily Living
i-ADL DI: Instrumental Activities of Daily Living Disability Index
ICF: International Classification of Functioning Disability and Health
IQR: Interquartile Range
LAWTON: Lawton Instrumental Activities of Daily Living Scale
MCI: Mild Cognitive Impairment
MMSE: Mini-Mental State Examination
NINDS-ADRDA: National Institute of Neurological and Communicative Disorders and Stroke – Alzheimer’s Disease and Related Disorder Association
SD: Standard Deviation
TMT-A: Trail Making Test part A
TMT-B: Trail Making Test part B
VIF: Variance Inflation Factor
