# Supplementary material for: Influencing factors on instrumental activities of daily living functioning in people with mild cognitive disorder – a secondary investigation of cross-sectional data
Source: BMC Geriatr. 2022 Oct 11;22:791. doi: 10.1186/s12877-022-03476-8 (PMC9552428; doi:10.1186/s12877-022-03476-8)
Supplement: Supplementary file 1 — Additional file 1. [file 12877_2022_3476_MOESM1_ESM.pdf]

```
##Modellvalidation May 2022


rm(list=ls())
options(scipen=999)
library(psych)
library(data.table)
library(ggplot2)
library(lmtest)
library(car)


##Read in
data-----------------------------------------------------------------------------------------
d.val0<-
read.table("data_clean.csv",sep=",",header=TRUE,na.strings=c(NA,"99","999"),
stringsAsFactors = TRUE)
data.frame(names(d.val0))
str(d.val0)
summary(d.val0)
describe(d.val0)

##Between group tests
demographics---------------------------------------------------------------------------------
d.valS<-split(d.val0[,c(2,3,4,5,6,7,13,14,15)],f=d.val0$Diagnosis)
lapply(d.valS,describe)
lapply(d.valS,summary)
t.test(age~Diagnosis,d.val0)
t.test(edu~Diagnosis,d.val0)
t.test(comorbidities~Diagnosis,d.val0)
t.test(MMSE~Diagnosis,d.val0)
t.test(CAMCOGTot~Diagnosis,d.val0)
t.test(comorbidities~Diagnosis,d.val0)
t.test(children~Diagnosis,d.val0)
cross<-table(d.val0$sex,d.val0$Diagnosis)

chisq.test(cross, simulate.p.value = TRUE)
cross2<-table(d.val0$living,d.val0$Diagnosis)
chisq.test(cross2, simulate.p.value=TRUE)

##Between group tests
predictors-----------------------------------------------------------------------------------
d.valP<-
split(d.val0[,c(8,9,10,11,12,13,15,16,17,18,19,20,21)],f=d.val0$Diagnosis)
lapply(d.valP,describe)
lapply(d.valP,summary)
t.test(IADL~Diagnosis, d.val0)
t.test(mobility~Diagnosis, d.val0)
t.test(TINETTI~Diagnosis, d.val0)
t.test(memoryadas~Diagnosis, d.val0)
t.test(memoryCam~Diagnosis, d.val0)
t.test(attentionCAM~Diagnosis, d.val0)
t.test(attentionTMTA~Diagnosis, d.val0)
t.test(exefunTMTB~Diagnosis, d.val0)
t.test(exefunFAB~Diagnosis, d.val0)
cross<-table(d.val0$vision,d.val0$Diagnosis)
chisq.test(cross, simulate.p.value = TRUE)
cross<-table(d.val0$auditory,d.val0$Diagnosis)
```

```
chisq.test(cross, simulate.p.value = TRUE)
cross<-table(d.val0$living,d.val0$Diagnosis)
chisq.test(cross, simulate.p.value = TRUE)

##Inspect Distributions and
Correlations-------------------------------------
plot(d.val0$IADL)
hist(d.val0$IADL)
plot(d.val0$vision)
plot(d.val0$auditory)
plot(d.val0$mobility)
hist(d.val0$mobility)
plot(d.val0$TINETTI)
hist(d.val0$TINETTI)
plot(d.val0$living)
plot(d.val0$edu)
plot(d.val0$memoryadas)
hist(d.val0$memoryadas)
plot(d.val0$memoryCam)
hist(d.val0$memoryCam)
plot(d.val0$attentionCAM)
hist(d.val0$attentionCAM)
plot(d.val0$attentionTMTA)
hist(d.val0$attentionTMTA)
plot(d.val0$exefunTMTB)
hist(d.val0$exefunFAB)
scatterplotMatrix(~IADL+mobility+TINETTI+edu+memoryadas+memoryCam+
                    attentionCAM+attentionTMTA+exefunTMTB+exefunFAB,
                    data = d.val0, smooth=FALSE)
scatterplotMatrix(~IADL+vision+auditory+living, data =d.val0, smooth =
FALSE)

d.val2<-d.val0[,c(9,10,11,12,13,14,15,16,17,18,19,20,21)]
str(d.val2)
d.val2[,c(1,2,5)]<-lapply(d.val2[,c(1,2,5)],function(x)as.numeric(x))
cor(d.val2,use = "pairwise", method = "pearson")

#MULTIVARIATE ANLAYSIS
lm(IADL~vision, data = d.val0)
lm(IADL~auditory, data = d.val0)
lm(IADL~mobility, data = d.val0)
lm(IADL~TINETTI, data = d.val0)
lm(IADL~living, data = d.val0)
lm(IADL~edu, data = d.val0)
lm(IADL~memoryadas, data = d.val0)
lm(IADL~memoryCam, data = d.val0)
lm(IADL~attentionCAM, data = d.val0)
lm(IADL~attentionTMTA, data = d.val0)
lm(IADL~exefunTMTB, data = d.val0)
lm(IADL~exefunFAB, data = d.val0)
form<-formula(IADL~vision+auditory+mobility+TINETTI+living+edu



              +memoryadas+memoryCam+attentionCAM+attentionTMTA+exefunTMTB
              +exefunFAB)
mf<-model.frame(form,d.val0,na.action=na.pass)
str(mf)
#mf$IADL<-(mf$IADL*(105-1)+0.5)/105
md.pattern(mf,rotate=TRUE, plot=TRUE)
```

```
describe(mf)

modLinmax<-lm(IADL~vision+auditory+mobility+TINETTI+living
              +edu+memoryadas+memoryCam+attentionCAM+attentionTMTA
              +exefunTMTB+exefunFAB,data=mf)
mf<-model.frame(modLinmax, data = mf)
str(mf)
describe(mf)
summary(modLinmax)

##Selection of predictors based on multiple regression models
modLinmin1<-lm(IADL~vision+auditory+mobility+TINETTI+living+edu
               +memoryCam+attentionTMTA+exefunTMTB, data = mf)
summary(modLinmin1)

s2<-summary(modLinmin1)$coef
s2
modLinmin2<-lm(IADL~vision+auditory+mobility+TINETTI+living+edu
               +memoryadas+attentionCAM+exefunTMTB,data=mf)
summary(modLinmin2)
modLinmin3<-lm(IADL~vision+auditory+mobility+TINETTI+living+edu
               +memoryadas+attentionCAM+exefunFAB,data=mf)
summary(modLinmin3)
modLinmin4<-lm(IADL~vision+auditory+mobility+TINETTI+living+edu
               +memoryCam+attentionTMTA+exefunFAB,data=mf)
summary(modLinmin4)
modLinmin5<-lm(IADL~vision+auditory+mobility+TINETTI+living+edu
               +memoryCam+attentionCAM+exefunFAB,data=mf)
summary(modLinmin5)
modLinmin6<-lm(IADL~vision+auditory+mobility+TINETTI+living+edu
               +memoryCam+attentionCAM+exefunTMTB,data=mf)
summary(modLinmin6)
modLinmin7<-lm(IADL~vision+auditory+mobility+TINETTI+living+edu
               +memoryadas+attentionTMTA+exefunFAB,data=mf)
summary(modLinmin7)
modLinmin8<-lm(IADL~vision+auditory+mobility+TINETTI+living+edu
               +memoryadas+attentionTMTA+exefunTMTB,data=mf)
summary(modLinmin8)

AIC(modLinmin1)
AIC(modLinmin2)
AIC(modLinmin3)
AIC(modLinmin4)
AIC(modLinmin5)
AIC(modLinmin6)
AIC(modLinmin7)
AIC(modLinmin8)


summary(modLinmin8)
confint(modLinmin8)

##Model Diagnostics
#Residuals
plot(modLinmin8)
#Unusual datapoints
cooks.distance(modLinmin8)
4/(105-9-1)
```

```
#Heteroscedasticity
bptest(modLinmin8)
#Multicollinearity
vif(modLinmin8)

##Weighting of predictors
mf[,c(2,3,6)]<-lapply(mf[,c(2,3,6)],function(x)as.numeric(x)-1)
mod<-lm(IADL~vision+auditory+mobility+TINETTI+living+edu
         +memoryadas+attentionTMTA+exefunTMTB,data=mf)
summary(mod)$coef
modstand<-lm(scale(IADL)~scale(vision)+scale(auditory)+scale(mobility)


+scale(TINETTI)+scale(living)+scale(edu)
              +scale(memoryadas)+scale(attentionTMTA)
+scale(exefunTMTB),data=mf) #SD change pro SD change
summary(modstand)
mod<-lm(IADL~vision+auditory+TINETTI+living+edu
        +memoryadas+attentionTMTA+exefunTMTB,data=mf)
summary(mod)$coef
effectsize::standardize_parameters(mod)

s2<-summary(modstand)$coef

str(s2)
s2<-data.frame(s2[-1,])
str(s2)
ggplot(data=s2) +
  geom_bar( aes(x=row.names(s2), y=abs(s2$Estimate)),stat="identity",
fill="skyblue", alpha=.9) +
  geom_errorbar( aes(x=row.names(s2), ymin=abs(s2$Estimate)-s2$Std..Error,
ymax=abs(s2$Estimate)+s2$Std..Error), width=0.4, colour="orange",
alpha=0.9, size=1.3)+
 coord_flip()+xlab("Variable")+ylab("Weight")


ggplot(data=s2) +
  geom_bar( aes(x=row.names(s2), y=abs(Estimate)),stat="identity",
fill="darkgrey", alpha=.95) +
  geom_errorbar( aes(x=row.names(s2), ymin=abs(Estimate)-Std..Error,
ymax=abs(Estimate)+Std..Error), width=0.4, colour="black", alpha=0.9,
size=1)+
 coord_flip()+xlab("Variable")+ylab("Effect")







##Subgroup Analysis MCI

d.val1<-subset(d.val0,Diagnosis=="MCI")
str(d.val1)
```

```
describe(d.val1)

form<-formula(IADL~vision+auditory+mobility+TINETTI+living+edu
              +memoryadas+memoryCam+attentionCAM+attentionTMTA+exefunTMTB
              +exefunFAB)

mf<-model.frame(form,d.val1,na.action=na.pass)
str(mf)
#mf$IADL<-(mf$IADL*(64-1)+0.5)/64
md.pattern(mf,rotate=TRUE)
describe(mf)


modLinminMCI2<-lm(IADL~vision+auditory+mobility+TINETTI+living+edu

                 +memoryadas+attentionTMTA+exefunTMTB,data=mf)
summary(modLinminMCI2, conf.int = TRUE)
confint(modLinminMCI2)
effectsize::standardize_parameters(modLinminMCI2)
##Modeldiagnostics
#Residuals
plot(modLinminMCI2)

#Unusual datapoints
cooks.distance(modLinminMCI2)
4/(64-9-1)

#Heteroscedasticity
bptest(modLinminMCI2)
#Multicollinearity
vif(modLinminMCI2)
```
