# Supplementary material for: Influencing factors on instrumental activities of daily living functioning in people with mild cognitive disorder – a secondary investigation of cross-sectional data
Source: BMC Geriatr. 2022 Oct 11;22:791. doi: 10.1186/s12877-022-03476-8 (PMC9552428; doi:10.1186/s12877-022-03476-8)
Supplement: Supplementary file 2 — Additional file 2: Table 1. Pairwise Inter-variable correlations. [file 12877_2022_3476_MOESM2_ESM.docx]

| Additional Table 1 – Pairwise Inter-variable correlations | | | | | | | | | | | | |
| --- | --- | --- | --- | --- | --- | --- | --- | --- | --- | --- | --- | --- |
|  | vision | auditory | mobility | balance^*^ | living | edu-cation | memoryadas | memoryCAM | attentionCAM | TMTA | TMTB | FAB |
| vision | 1.000 | 0.173 | 0.135 | -0.308 | -0.074 | 0.045 | 0.012 | -0.002 | -0.091 | 0.189 | 0.322 | -0.099 |
| auditory |  | 1.000 | 0.087 | -0.052 | -0.049 | -0.032 | 0.003 | 0.111 | -0.081 | -0.116 | -0.193 | -0.029 |
| mobility |  |  | 1.000 | -0.691 | -0.245 | -0.184 | 0.021 | 0.026 | -0.188 | -0.019 | 0.149 | -0.286 |
| balance^*^ |  |  |  | 1.000 | 0.262 | 0.124 | 0.117 | -0.005 | 0.070 | -0.199 | -0.215 | 0.330 |
| living |  |  |  |  | 1.000 | 0.052 | -0.079 | -0.115 | 0.073 | 0.044 | 0.059 | -0.051 |
| education |  |  |  |  |  | 1.000 | -0.07 | 0.198 | 0.210 | -0.225 | -0.245 | 0.345 |
| memoryadas |  |  |  |  |  |  | 1.000 | 0.390 | 0.077 | -0.290 | -0.354 | 0.087 |
| memoryCAM |  |  |  |  |  |  |  | 1.000 | 0.031 | -0.382 | -0.408 | 0.129 |
| attentionCAM |  |  |  |  |  |  |  |  | 1.000 | -0.124 | 0.031 | 0.143 |
| TMTA |  |  |  |  |  |  |  |  |  | 1.000 | 0.530 | -0.407 |
| TMTB |  |  |  |  |  |  |  |  |  |  | 1.000 | -0.022 |
| FAB |  |  |  |  |  |  |  |  |  |  |  | 1.000 |
| Abbreviations: memoryadas, Memory Subscale Alzheimer’s Disease Assessment Scale; memoryCAM, Memory Subscale Cambridge Cognitive Test-Revised; attentionCAM, attention subscale Cambridge Cognitive Test-Revised; TMTA, Trail Making Test part A; TMTB, Trail Making Test part B; FAB, Frontal Assessment Battery  ^*^ balance assessed with Tinetti Test | | | | | | | | | | | | |
