# Supplementary material for: Influencing factors on instrumental activities of daily living functioning in people with mild cognitive disorder – a secondary investigation of cross-sectional data
Source: BMC Geriatr. 2022 Oct 11;22:791. doi: 10.1186/s12877-022-03476-8 (PMC9552428; doi:10.1186/s12877-022-03476-8)
Supplement: Supplementary file 3 — Additional file 3: Table 2. Cook’s Distance (Cook’s D) of the model including complete cases of participants with aMCI and mild dementia. Table 3. Variance Inflation Factor (VIF) coefficient estimates of the predictors including complete cases of participants with aMCI and mild dementia. Table 4. Cook’s Distance (Cook’s D) of the model including complete cases of participants with a-MCI. Table 5. Results of studentized Breusch-Pagan test model including complete cases of participants with a-MCI. Table 6. Variance Inflation Factor (VIF) coefficient estimates of the predictors including complete cases of participants with a-MCI. Figure 1. Residual versus Fitted values, model including complete cases of participants with aMCI and mild dementia. Figure 2. Normal Q-Q Plot, model including complete cases of participants with aMCI and mild AD. Figure 3. Scale Location, model including complete cases of participants with aMCI and mild AD. Figure 4. Residual versus Fitted values, model including complete cases of participants with a-MCI. Figure 5. Normal Q-Q Plot, model including complete cases of participants with a-MCI. Figure 6. Scale Location, model including complete cases of participants with a-MCI. [file 12877_2022_3476_MOESM3_ESM.docx]

**Model Diagnostics**

| Additional Table 2 - Cook’s Distance (Cook’s D) of the model including complete cases of participants with aMCI and mild dementia | | | | | | | | | |
| --- | --- | --- | --- | --- | --- | --- | --- | --- | --- |
| ID | 1 | 5 | 7 | 8 | 9 | 10 | 14 | 17 | 18 |
| Cook’s D | 0.030 | 0.014 | 0.046 | 0.001 | 0.021 | 0.001 | 0.040 | 0.118 | 0.000 |
| ID | 20 | 24 | 26 | 29 | 30 | 31 | 33 | 35 | 39 |
| Cook’s D | 0.000 | 0.090 | 0.012 | 0.003 | 0.001 | 0.003 | 0.000 | 0.016 | 0.003 |
| ID | 40 | 41 | 45 | 46 | 47 | 49 | 51 | 52 | 55 |
| Cook’s D | 0.004 | 0.008 | 0.043 | 0.037 | 0.022 | 0.057 | 0.223 | 0.013 | 0.003 |
| ID | 58 | 63 | 66 | 69 | 71 | 72 | 78 | 82 | 88 |
| Cook’s D | NaN | 0.001 | 0.060 | 0.013 | 0.029 | 0.102 | 0.000 | 0.001 | 0.004 |
| ID | 99 |  |  |  |  |  |  |  |  |
| Cook’s D | 0.033 |  |  |  |  |  |  |  |  |
| Cook’s D > 4 / (105-9-1) indicate unusual data points | | | | | | | | | |

| Additional Table 3 - Variance Inflation Factor (VIF) coefficient estimates of the predictors including complete cases of participants with aMCI and mild dementia | | | | | | | | | |
| --- | --- | --- | --- | --- | --- | --- | --- | --- | --- |
| **Predictor** | vision | auditory | mobility | TINETTI | living | education | ADAS | TMTA | TMTB |
| **VIF** | 1.766 | 1.597 | 1.680 | 1.608 | 1.163 | 1.330 | 1.537 | 1.660 | 2.000 |
| Abbreviations: ADAS, Memory Subscale of the ADAS-cog; TMTA, Trail Making Test part A; TMTB, Trail Making Test part B | | | | | | | | | |

| Additional Figure 1 – Residual versus Fitted values, model including complete cases of participants with aMCI and mild dementia |
| --- |
| 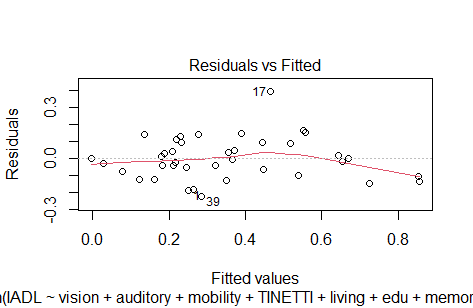 |
| Diagnostic plot to check the linear relationship assumption, a non-horizontal line with a distinct pattern would indicate a violation of the model assumption |

| Additional Figure 2 – Normal Q-Q Plot, model including complete cases of participants with aMCI and mild AD |
| --- |
| 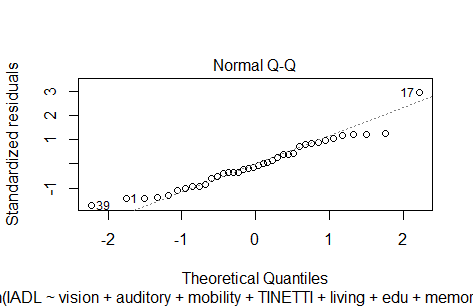 |
| Diagnostic plot to examine normal distribution of residuals. Residuals should follow the straight dashed line. |

| Additional Figure 3 – Scale Location, model including complete cases of participants with aMCI and mild AD |
| --- |
| 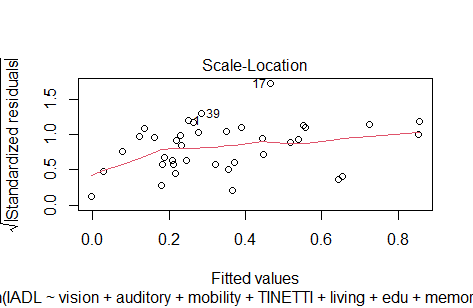 |
| Diagnostic plot to examine homoscedasticity. A horizontal line with equally spread points indicates homoscedasticity. |

**Model diagnostics subgroup analysis, participants with a-MCI**

| Additional Table 4 - Cook’s Distance (Cook’s D) of the model including complete cases of participants with a-MCI | | | | | | | | | |
| --- | --- | --- | --- | --- | --- | --- | --- | --- | --- |
| ID | 1 | 5 | 7 | 8 | 9 | 10 | 14 | 17 | 18 |
| Cook’s D | 0.029 | 0.881 | 0.042 | 0.000 | 0.032 | 0.003 | 0.037 | 0.180 | 0.002 |
| ID | 20 | 24 | 26 | 29 | 30 | 31 | 33 | 35 | 39 |
| Cook’s D | 0.009 | 0.301 | 0.009 | 0.003 | 0.001 | 0.003 | 0.000 | 0.017 | 0.033 |
| ID | 40 | 41 | 45 | 46 | 47 | 49 | 51 | 52 | 55 |
| Cook’s D | 0.018 | 0.011 | 0.041 | 0.025 | 0.016 | 0.014 | 0.881 | 0.010 | 0.008 |
| ID | 58 | 63 |  |  |  |  |  |  |  |
| Cook’s D | NaN | 0.001 |  |  |  |  |  |  |  |
| Cook’s D > 4 / (65-9-1) indicate unusual data points | | | | | | | | | |

| Additional Table 5 - Results of studentized Breusch-Pagan test model including complete cases of participants with a-MCI |
| --- |
| BP = 4.0823, df = 9, p-value = 0.9059 |
|  |

| Additional Table 6 - Variance Inflation Factor (VIF) coefficient estimates of the predictors including complete cases of participants with a-MCI | | | | | | | | | |
| --- | --- | --- | --- | --- | --- | --- | --- | --- | --- |
| **Predictor** | vision | auditory | mobility | TINETTI | living | education | ADAS | TMTA | TMTB |
| **VIF** | 2.268 | 1.804 | 1.758 | 1.895 | 1.185 | 1.630 | 1.163 | 1.353 | 1.900 |
| Abbreviations: ADAS, Memory Subscale of the ADAS-cog; TMTA, Trail Making Test part A; TMTB, Trail Making Test part B | | | | | | | | | |

| Additional Figure 4 – Residual versus Fitted values, model including complete cases of participants with a-MCI |
| --- |
| 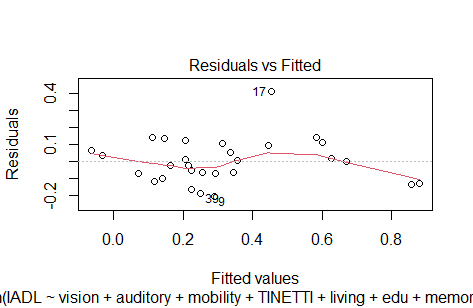 |
| Diagnostic plot to check the linear relationship assumption, a non-horizontal line with a distinct pattern would indicate a violation of the model assumption |

| Additional Figure 5 – Normal Q-Q Plot, model including complete cases of participants with a-MCI |
| --- |
| 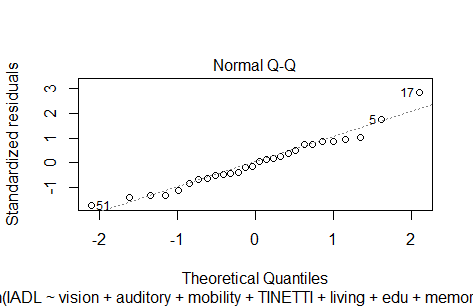 |
| Diagnostic plot to examine normal distribution of residuals. Residuals should follow the straight dashed line. |

| Additional Figure 6 – Scale Location, model including complete cases of participants with a-MCI |
| --- |
| 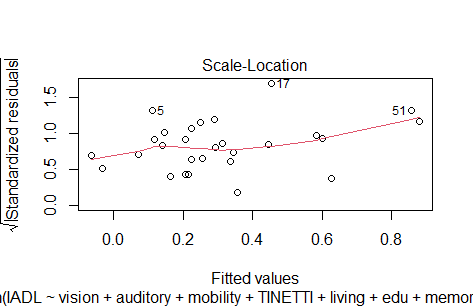 |
| Diagnostic plot to examine homoscedasticity. A horizontal line with equally spread points indicates homoscedasticity. |
